# Patients with overlapping diagnoses of asthma and COPD: is livestock exposure a risk factor for comorbidity and coexisting symptoms and infections?

**DOI:** 10.1186/s12890-019-0865-z

**Published:** 2019-06-10

**Authors:** Christos Baliatsas, Lidwien A. M. Smit, Michel L. A. Dückers, Christel E. van Dijk, Dick Heederik, C. Joris Yzermans

**Affiliations:** 1 Netherlands Institute for Health Services Research (NIVEL), Otterstraat 118-124, 3513 CR Utrecht, The Netherlands; 2 Institute for Risk Assessment Sciences (IRAS), Utrecht University, Utrecht, The Netherlands

**Keywords:** Livestock, Environmental exposure, ACOS, COPD, Asthma, Epidemiology

## Abstract

**Background:**

Epidemiological research on health effects of livestock exposure in population subgroups with compromised respiratory health is still limited. The present study explored the association between livestock exposure and comorbid/concurrent conditions in patients with overlapping diagnoses of asthma and COPD.

**Methods:**

Electronic health record data from 23 general practices in the Netherlands were collected from 425 patients diagnosed with both asthma and COPD, living in rural areas with high livestock density (“study area”). Data of 341 patients with the same overlapping diagnoses, living in rural areas with lower livestock density (“control areas”) were obtained from 19 general practices. First, the prevalence of comorbid disorders and symptoms/infections were compared between the study and control area. Second, the examined health outcomes were analyzed in relation to measures of individual livestock exposure.

**Results:**

Pneumonia was twice as common among patients living in areas with a high livestock density (OR 2.29, 99% CI 0.96–5.47); however, there were generally no statistically significant differences in the investigated outcomes between the study and control area. Significant associations were observed between presence of goats within 1000 m and allergic rhinitis (OR 5.71, 99% CI 1.11–29.3, *p* < 0.01), number of co-occurring symptoms (IRR 1.69, 99% CI 1.03–2.77, p < 0.01) and anxiety (OR 8.18, 99% 1.5–44.7, p < 0.01). Presence of cattle within 500 m was associated with pneumonia prevalence (OR 2.48, 99% CI 1.05–5.84, p < 0.01).

**Conclusion:**

Livestock exposure is not associated with comorbid chronic conditions but appears to be a risk factor for symptomatic effects in patients with overlapping diagnoses of asthma and COPD.

## Background

Patients with obstructive lung diseases such as asthma and chronic obstructive pulmonary disease (COPD) are characterized by substantial morbidity. Among the different phenotypes that have been identified for asthma and COPD [1–3], the largest one represents a patient group that has features and characteristics of both diseases [4, 5]. Overlapping diagnoses of COPD and asthma is commonly referred to as Asthma and COPD Overlap Syndrome (ACOS) [6]. The health-care burden of ACOS seems to be considerable; recent studies suggest that, compared to COPD or asthma only, ACOS patients have higher comorbidity and exacerbation rates, lower health-related quality of life and make more often use of healthcare [7–9].

It is well documented that particulate matter and its components contribute to the development and exacerbation of respiratory diseases [10–12]. Individuals with asthma for instance suffer more exacerbations and they appear to be at high risk of developing ACOS when exposed to air pollution [13, 14]. Livestock farming is one of the continuously expanding sources of ambient air pollution worldwide [15, 16]. In the international literature different population-based studies have described the relationship between livestock farming and health effects such as asthma, allergy, respiratory complaints, pneumonia, and lung function deficits [17–25].

Fewer studies have focused on specific respiratory patient groups, although these seem to respond to a greater extent to exposure from livestock farms. In an experimental setting, the study of Sigurdarson et al. [26] demonstrated adverse reactions in individuals with asthma after being exposed to inhaled grain dust and Harting et al. [27] found that COPD patients had greater systemic responsiveness to ex vivo stimulation with swine dust extract than healthy volunteers. In a more recent epidemiological study in the Netherlands, it was shown that COPD patients living at shorter distance from livestock farms reported more respiratory symptoms [28] and had a higher exacerbation rate when compared with a control area in COPD [29]; however, no consistent associations were observed between individual exposure estimates and diagnosed comorbidity or exacerbations in COPD patients [29, 30].

Considering the growing evidence, it seems important to lay more emphasis on investigating whether the effects of livestock exposure on morbidity and symptomatic reactions are more pronounced among respiratory patients with other clinical phenotypes. To date, no epidemiological study has been conducted on the association between health outcomes and livestock density in patients with ACOS. The present study uses electronic health record data from general practices to address the following questions: 1) Does the prevalence of the examined health outcomes in those patients differ between areas with high livestock farm density (“study areas”) and rural areas with a substantially lower livestock density (“control areas”); 2) What is the association between indicators of exposure to livestock farms and comorbidities and symptoms and infections in ACOS patients? Following prior findings on other respiratory vulnerable subgroups, it is hypothesized that a) the prevalence of the investigated health outcomes will be higher in the “exposed” area; b) higher individual exposure, as indicated by the employed proxies, will be more consistently associated with higher prevalence of comorbid conditions and symptoms.

## Methods

### Study design and population

This study is part of a larger study in the Netherlands, entitled “Livestock Farming and Neighbouring Residents’ Health study” (VGO). Patients were sampled from 23 general practices in rural areas with high livestock farm density (eastern part of Noord-Brabant province as well as the northern part of Limburg); more than 95% of the included patients were living within a km from an animal farm. General practices in the “control area” (*n* = 19) were located in other rural areas in the Netherlands with a substantially lower livestock farm density (control area) [29, 31], particularly in the provinces of Noord-Holland, Zuid- Holland, Utrecht, Gelderland, Zeeland, Overijssel and Groningen (for additional details, see van Dijk et al., 2017 [31]).

Diagnosed conditions were registered by the general practitioners (GPs) on the basis of the International Classification of Primary Care (ICPC) [32]. For the present analysis, 766 (*n* = 425 in the “study area” and *n* = 341 in the control group) ACOS patients (registered with ICPC codes R95 or R91: COPD and R96: Asthma) with an age of ≥40 years were included. The study focused on non-occupational exposure, therefore patients possibly working or residing on a farm (defined as a distance of < 50 m between house address and the closest farm) were not included. Additional information regarding study design is presented in previous publications [29, 31].

### Health outcome assessment

**Fig. 1:**
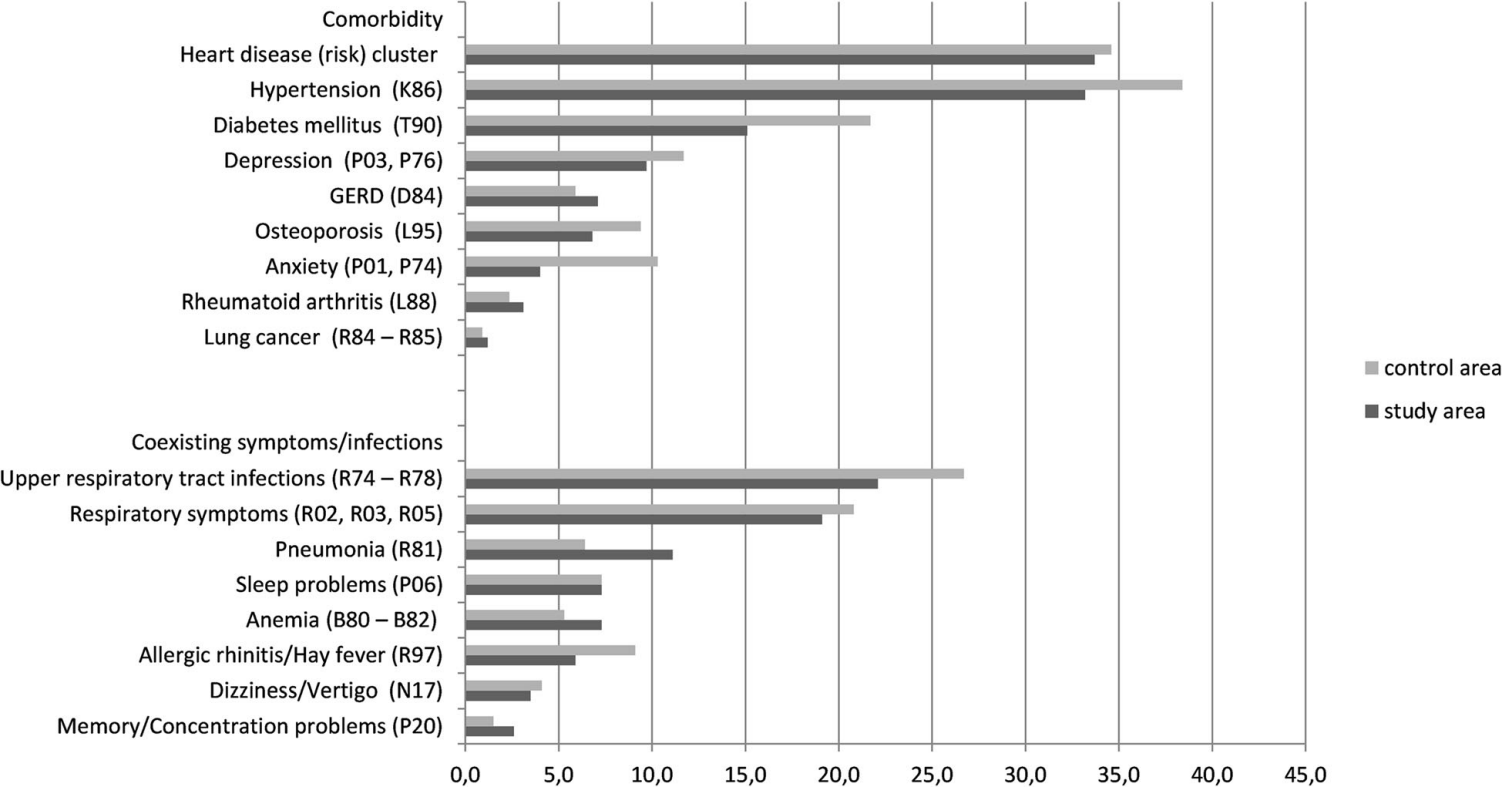
Prevalence (and corresponding ICPC codes) of comorbid conditions and coexisting symptoms and infections in the sample of ACOS patients living in the study area (*n* = 425) and control area (*n* = 341)**.** Abbreviations: ICPC; International Classification of Primary Care; GERD; Gastro-oesophageal reflux disease. * The heart disease (risk) cluster consists of coronary heart disease, heart failure, atherosclerosis, hyperlipidemia

Data from electronic health records (EHRs) from primary care (general practices) were used [33]. These practices participated in the Primary Care Database (PCD) of NIVEL [33, 34]. Prevalence rates of the year 2012 were estimated [30]. Contacts for the same health problem within a specific time frame were clustered into episodes of care, which were used to estimate the prevalence rates. For the construction of care episodes, all records with an ICPC code were considered [31, 34]. Selection of comorbid conditions (Fig. 1) was based on the literature [9, 35–38].

### Exposure indicators

For the same year (2012), data regarding characteristics of farms was obtained from databases of environmental licenses for livestock (“Bestand Veehouderij Bedrijven”). These include information on animal type and number, geographic coordinates of farms and fine dust and ammonia (NH_3_) emission estimates from each farm per year. The distance between home addresses and farms was estimated based on a geographic information system (GIS) (ArcGis 9.3.1, Esri, Redlands, CA). Similar to recent studies [28–31] we examined the following variables: 1) distance (meters) from participant’s home to the nearest animal farm (binary) and 2) presence of farm animals (various types) within 500 m and 1000 m (binary). In additional analyses, inverse-distance weighted fine dust and NH_3_ emissions from all farms within 500 m and 1000 m were also analyzed, as continuous variables. These were rescaled using interquartile range (IQR) as a scaling factor.

### Statistical analysis

The prevalence of morbidity rates and coexisting symptoms in the sample as well as basic sample characteristics were estimated on the basis of descriptive analyses. Given that the study data have a hierarchical structure (observations clustered in general practices), exposure-outcome associations were examined using multilevel logistic and Poisson regression models. Analyses were adjusted for gender and age (including a quadratic variable to allow for a potential non-linear trend between age and morbidity). For each regression model, Odds ratios (OR) for the binary outcomes and incidence rate ratios for the count variables (IRR) and 99% confidence intervals (CI) were calculated. Statistical significance was set at *p* < 0.01, to correct for multiple testing. Analyses were performed with STATA version 13.0 (StataCorp LP, College Station, TX, USA).

## Results

### Descriptive analysis

An overview of sample characteristics is shown in Table 1. Patients with ACOS in the study area were slightly more often female (52%) and were on average 66 years of age. At least one comorbid condition was present in 67% of the patients while about 54% had at least one coexisting symptom/infection. About 50% of the patients in the study area lived within 500 m from an animal farm. Patients in the study area were also frequently living within 500 m of farms with cattle (31%) and pigs (19%). These rates were much higher within 1000 m (Table 1).

**Table 1:** Characteristics of ACOS patients included in the study

Characteristic	Study area (*n* = 425)	Control area (*n* = 341)
*Demographics*		
Female gender (%)	52.2	59.5
Mean age (SD)	66.4 (11.4)	67.1 (12.4)
*Exposure*		
Distance to the nearest farm (%)		
< 250 m	15.1	
250 – 500 m	32.5	
Presence of farm animals within 500 m (%)		
Mink	1.9	
Poultry	9.7	
Pigs	19.1	
Goats^c^	0.2	
Cattle	31.1	
Presence of farm animals within 1000 m (%)		
Mink	10.8	
Poultry	46.6	
Pigs	79.1	
Goats	5.7	
Cattle	87.8	
*Comorbidity*		
Total prevalence (%)^a^	67.3	67.7
Mean number (SD)^b^	1.14 (1.03)	1.35 (1.26)
*Coexisting symptoms/infections*		
Total prevalence (%)^a^	54.1	52.8
Mean number (SD)^b^	0.79 (0.88)	0.81 (0.95)

Abbreviations: *SD* Standard deviation

^a^Patients with at least one of the investigated outcomes

^b^Count variable, expressed in mean score

^c^Excluded from the main analyses due to the small number of cases

As shown in Fig. 1, among the examined chronic conditions, heart disease (study group 33.7% vs. control group 34.6%), hypertension (33.2% vs. 38.4%), diabetes (15.1% vs. 21.7%) and depression (9.7% vs. 11.7%) were the most prevalent in both the study and control group. Upper respiratory tract infections (22.1% vs. 26.7%), respiratory symptoms (19.1% vs. 20.8%), pneumonia (11.1% vs. 6.5%) and sleep problems (7.3% vs. 7.3%) were the most prevalent acute conditions.

### Comorbid diseases and symptoms: comparison between study and control area

No significant differences were found in the majority of examined chronic conditions; in some cases (e.g. diabetes) prevalence was lower in the study area (Table 2). Higher but nonsignificant OR were observed for several coexisting symptoms/infections such as pneumonia (OR 2.29, 99% CI 0.96–5.47) and also for the total symptom/infection prevalence (OR 1.28, 99% CI 0.71–2.30) (Table 2).

**Table 2:** Differences (OR, 99% CI) ^a^ in prevalence of comorbidity and coexisting symptoms and infections, between ACOS patients living in the study area and those living in the control area (significant associations in bold)

	Study area vs. control area (OR, 99% CI)
Comorbidity	
GERD	1.25 (0.58–2.7)
Osteoporosis	0.76 (0.27–2.17)
Diabetes mellitus	0.6 (0.37–0.99)*
Anxiety	0.41 (0.16–1.04)
Depression	0.85 (0.44–1.62)
Lung cancer	1.09 (0.11–10.7)
Hypertension	0.94 (0.54–1.64)
Rheumatoid arthritis	1.34 (0.41–4.37)
Heart disease (risk) cluster^b^	0.84 (0.47–1.51)
Total prevalence^c^	1.02 (0.63–1.65)
Number^d^	0.86 (0.69–1.05)
Coexisting symptoms & infections	
Pneumonia	2.29 (0.96–5.47)
Sleep problems	1.16 (0.37–3.64)
Memory/Concentration problems	1.93 (0.36–10.2)
Upper respiratory tract infections	1.01 (0.46–2.22)
Respiratory symptoms	0.99 (0.55–1.79)
Dizziness/Vertigo	0.85 (0.32–2.27)
Anemia	1.52 (0.68–3.41)
Allergic rhinitis/Hay fever	0.62 (0.28–1.42)
Total prevalence^c^	1.28 (0.71–2.3)
Number^d^	1.11 (0.8–1.55)

Abbreviations: *ORs* Odds ratios, *CI* Confidence intervals, *GERD* Gastro-oesophageal reflux Disease

^a^Adjusted for age and gender

^b^The heart disease (risk) cluster consists of coronary heart disease, heart failure, atherosclerosis hyperlipidemia

^c^Patients with at least one of the investigated outcomes

^d^Count variables, incidence rate ratios (IRR) are provided

*p < 0.01

### Association of livestock exposure with comorbid chronic conditions

As shown in Table 3, analyses yielded no significant associations between distance to the nearest farm and the investigated outcomes. Only presence of goats within 1000 m was significantly associated with a higher prevalence of anxiety (Table 5). There were no significant findings in relation to modeled fine dust and ammonia emissions (see Appendix).

**Table 3:** Association (OR, 99% CI) ^a^ between distance to the nearest farm and primary outcomes among ACOS patients in the study area

Comorbidity	< 250 m^b^	250 – 500 m^b^
GERD	0.94 (0.21–4.13)	1.16 (0.41–3.33)
Osteoporosis	0.42 (0.05–3.37)	1.4 (0.45–4.35)
Diabetes mellitus	1.28 (0.43–3.41)	0.86 (0.39–1.89)
Anxiety	1.19 (0.2–6.9)	1.32 (0.32–5.34)
Depression	0.35 (0.07–1.79)	0.82 (0.31–2.13)
Lung cancer	i.n.c	0.38 (0.02–6.94)
Hypertension	0.8 (0.34–1.89)	0.91 (0.49–1.69)
Rheumatoid arthritis	i.n.c	1.67 (0.35–7.92)
Heart disease (risk) cluster*	0.61 (0.27–1.38)	0.85 (0.46–1.58)
Total prevalence^c^	0.48 (0.21–1.12)	0.86 (0.44–1.65)
Number^d^	0.73 (0.5–1.07)	0.97 (0.75–1.25)
Coexisting symptoms & infections		
Pneumonia	2.11 (0.68–6.47)	1.73 (0.7–4.26)
Sleep problems	0.61 (0.11–3.36)	1.13 (0.38–3.32)
Memory/Concentration problems	i.n.c	i.n.c
Upper respiratory tract infections	0.8 (0.31–2.1)	0.84 (0.42–1.71)
Respiratory symptoms	0.78 (0.29–2.08)	1.02 (0.51–2.06)
Dizziness/Vertigo	0.38 (0.02–5.85)	0.95 (0.22–4.1)
Anemia	1.02 (0.23–4.58)	1.5 (0.54–4.19)
Allergic rhinitis/Hay fever	0.58 (0.07–4.63)	1.63 (0.48–5.47)
Total prevalence^c^	0.7 (0.32–1.52)	0.92 (0.52–1.66)
Number^d^	0.85 (0.55–1.31)	1.04 (0.76–1.41)

Abbreviations: *ORs* Odds ratios, *CI* Confidence intervals, *GERD* Gastro-oesophageal reflux disease, *i.n.c* Insufficient number of cases

^a^Adjusted for age and gender

^b^versus > 500 m (reference category)

^c^Patients with at least one of the investigated outcomes

^d^Count variables, incidence rate ratios (IRR) are provided

### Association between livestock exposure and coexisting symptoms/infections

Several significant associations were found between the examined exposure proxies and prevalence of co-occurring symptoms/infections. More specifically, presence of cattle within 500 m was associated with a higher prevalence of pneumonia (Table 4) and presence of goats within 1000 m with allergic rhinitis/hay fever and also with a number of co-occurring symptoms (Table 5). Overall, OR were consistently higher for most of the associations between presence of goat farms within 1000 m and acute conditions; a borderline nonsignificant association with pneumonia was also observed (OR 3.65, 99% CI 0.96–13.8). No statistically significant results were found in relation to distance to the nearest farm and modeled emissions but pneumonia risk was again, consistently higher (see Table 2 and Appendix). Analyses also showed a significant inverse association between pigs within 500 m and total prevalence (Table 4).

**Table 4:** Association (OR, 99% CI) ^a^ between presence of farm animals within 500 m and primary outcomes among ACOS patients in the study area (significant associations in bold)

Presence of farm animals within 500 m (yes/no)^d^				
Comorbidity	Mink	Poultry	Pigs	Cattle
GERD	4.6 (0.52–40.9)	1.47 (0.34–6.36)	1.3 (0.4–4.19)	0.93 (0.32–2.72)
Osteoporosis	i.n.c	1.13 (0.18–7.02)	1.22 (0.29–5.0)	0.91 (0.25–3.31)
Diabetes mellitus	2.26 (0.24–20.8)	1.63 (0.56–4.69)	1.15 (0.47–2.8)	1.05 (0.49–2.26)
Anxiety	i.n.c	i.n.c	0.56 (0.08–4.09)	1.61 (0.43–5.98)
Depression	i.n.c	1.64 (0.43–6.29)	1.1 (0.37–3.28)	0.59 (0.21–1.66)
Lung cancer	i.n.c	2.26 (0.12–42.7)	i.n.c	i.n.c
Hypertension	0.8 (0.09–7.31)	1.04 (0.41–2.66)	1.23 (0.6–2.53)	1.07 (0.57–2.02)
Rheumatoid arthritis	i.n.c	3.02 (0.49–18.5)	0.36 (0.02–5.67)	0.42 (0.05–3.2)
Heart disease (risk) cluster*	0.6 (0.07–5.1)	1.4 (0.55–3.59)	1.17 (0.57–2.41)	0.77 (0.42–1.41)
Total prevalence ^b^	0.47 (0.06–3.82)	1.14 (0.42–3.09)	1.02 (0.48–2.19)	0.78 (0.4–1.49)
Number ^c^	1.05 (0.44–2.51)	1.21 (0.84–1.75)	1.00 (0.73–1.35)	0.89 (0.68–1.15)
Coexisting symptoms & infections				
Pneumonia	1.4 (0.08–25.5)	1.16 (0.3–4.52)	0.76 (0.23–2.47)	2.48 (1.05–5.84)*
Sleep problems	i.n.c	0.72 (0.1–5.19)	0.96 (0.26–3.55)	1.28 (0.4–4.08)
Memory/Concentration problems	i.n.c	i.n.c	i.n.c	i.n.c
Upper respiratory tract infections	2.69 (0.36–19.8)	0.54 (0.16–1.84)	0.54 (0.21–1.39)	0.86 (0.41–1.77)
Respiratory symptoms	0.67 (0.04–11.0)	1.48 (0.54–4.1)	0.66 (0.26–1.65)	0.87 (0.43–1.77)
Dizziness/Vertigo	i.n.c	0.66 (0.04–9.88)	0.67 (0.09–4.92)	0.56 (0.1–3.05)
Anemia	i.n.c	1.88 (0.48–7.34)	1.06 (0.3–3.67)	1.1 (0.38–3.2)
Allergic rhinitis/Hay fever	i.n.c	0.89 (0.12–6.6)	0.17 (0.01–2.47)	1.53 (0.44–5.28)
Total prevalence ^b^	0.57 (0.08–4.07)	0.73 (0.3–1.77)	0.42 (0.21–0.84)*	0.74 (0.42–1.33)
Number^c^	0.84 (0.26–2.69)	0.97 (0.6–1.58)	0.66 (0.44–1.01)	1.03 (0.76–1.4)

^a^Adjusted for age and gender

^b^Patients with at least one of the investigated outcomes

^c^Count variables, incidence rate ratios (IRR) are provided

Abbreviations: *ORs* Odds ratios, *CI* Confidence intervals, *GERD* Gastro-esophageal reflux disease, *i.n.c* Insufficient number of cases

^d^Analyses in relation to goats were not feasible due to the small number of cases

*p < 0.01

**Table 5:** Association (OR, 99% CI) ^a^ between presence of farm animals within 1000 m and primary outcomes among ACOS patients in the study area (significant associations in bold)

Presence of farm animals within 1000 m (yes/no)					
Comorbidity	Mink	Poultry	Pigs	Goats	Cattle
GERD	1.79 (0.46–6.85)	1.51 (0.56–4.06)	0.57 (0.19–1.69)	1.02 (0.14–7.57)	0.62 (0.16–2.38)
Osteoporosis	0.61 (0.07–5.05)	0.87 (0.26–2.88)	2.57 (0.45–14.8)	0.52 (0.02–10.43)	1.56 (0.2–12.1)
Diabetes mellitus	1.33 (0.44–3.97)	0.92 (0.45–1.87)	0.85 (0.36–2.02)	0.48 (0.07–3.44)	1.3 (0.39–4.29)
Anxiety	1.08 (0.14–8.06)	0.66 (0.17–2.53)	0.3 (0.08–1.11)	8.18 (1.5–44.7) *	0.69 (0.12–3.79)
Depression	0.39 (0.05–2.68)	0.76 (0.31–1.84)	0.58 (0.22–1.49)	i.n.c	0.48 (0.16–1.41)
Lung cancer	i.n.c	0.28 (0.01–5.19)	0.82 (0.04–15.5)	i.n.c	i.n.c
Hypertension	1.14 (0.43–3.03)	0.87 (0.48–1.6)	1.11 (0.54–2.28)	1.49 (0.42–5.32)	1.18 (0.48–2.91)
Rheumatoid arthritis	i.n.c	1.35 (0.3–6.0)	4.11 (0.24–71.1)	1.72 (0.09–31.0)	1.82 (0.11–29.6)
Heart disease (risk) cluster*	1.41 (0.58–3.43)	0.61 (0.35–1.06)	1.31 (0.67–2.59)	1.24 (0.35–4.35)	1.21 (0.5–2.93)
Total prevalence^b^	1.11 (0.41–3.0)	0.56 (0.31–1.02)	0.98 (0.46–2.1)	1.77 (0.4–7.82)	1.03 (0.41–2.6)
Number^c^	1.11 (0.77–1.62)	0.87 (0.69–1.1)	0.95 (0.7–1.28)	1.15 (0.72–1.85)	1.1 (0.75–1.61)
Coexisting symptoms & infections					
Pneumonia	0.94 (0.2–4.32)	1.04 (0.42–2.58)	1.00 (0.35–2.86)	3.65 (0.96–13.8)	1.05 (0.27–4.04)
Sleep problems	0.87 (0.15–5.02)	0.56 (0.18–1.71)	2.23 (0.47–10.5)	1.92 (0.19–19.7)	1.39 (0.24–7.79)
Memory/Concentration problems	i.n.c	0.54 (0.09–3.19)	2.66 (0.15–45.8)	3.13 (0.22–43.2)	0.41 (0.04–3.95)
Upper respiratory tract infections	1.3 (0.43–3.98)	1.07 (0.55–2.11)	0.66 (0.3–1.44)	0.72 (0.15–3.4)	1.06 (0.39–2.88)
Respiratory symptoms	0.81 (0.26–2.49)	1.66 (0.87–3.19)	0.84 (0.39–1.81)	1.54 (0.42–5.62)	0.63 (0.25–1.58)
Dizziness/Vertigo	1.34 (0.18–10.0)	3.33 (0.72–15.4)	1.08 (0.2–5.93)	3.3 (0.41–26.5)	2.06 (0.14–30.6)
Anemia	0.83 (0.14–4.98)	1.04 (0.39–2.8)	0.78 (0.22–2.75)	1.75 (0.3–10.1)	0.45 (0.11–1.69)
Allergic rhinitis/Hay fever	0.38 (0.02–6.29)	1.16 (0.36–3.72)	2.54 (0.45–14.3)	5.71 (1.11–29.3) *	1.14 (0.2–6.43)
Total prevalence^b^	0.84 (0.34–2.09)	1.24 (0.71–2.18)	0.89 (0.44–1.8)	1.39 (0.41–4.63)	0.79 (0.34–1.84)
Number^c^	0.86 (0.52–1.41)	1.17 (0.88–1.55)	0.95 (0.67–1.37)	1.69 (1.03–2.77) *	0.86 (0.57–1.31)

Abbreviations: *ORs* Odds ratios, *CI* Confidence intervals, *GERD* Gastro-esophageal reflux disease, *i.n.c* Insufficient number of cases

^a^Adjusted for age and gender

^b^Patients with at least one of the investigated outcomes

^c^Count variables, incidence rate ratios (IRR) are provided

*p < 0.01

## Discussion

Asthma and COPD overlap syndrome is a recently recognized phenotype gaining attention due to the associated morbidity and rapid decline in lung function, compared to other respiratory conditions. While there is increasing evidence that livestock density can be an environmental risk factor for adverse effects in respiratory patients such as those with COPD [28, 29], its role in ACOS has not been previously investigated.

Determining the health effects of modifiable risk factors could decrease (multi)morbidity and burden of disease associated with ACOS. The present study comprises a first effort to fill this gap. General practice-registered patients with a dual diagnosis of asthma and COPD were included as a subgroup of respiratory patients potentially susceptible to livestock exposure. We used two approaches to assess the associations between exposure and various comorbid conditions and symptoms: First, we compared rural areas of high livestock density with other rural areas of substantially lower livestock density. Second, we tested the association between health outcomes and individual measures of livestock exposure, based on distance between patients’ home addresses and livestock farms.

In line with the broader literature, presence of comorbid conditions was found to be high in patients with ACOS [9, 37]. Overall, prevalence of the investigated outcomes did not differ significantly between the study and control area, but analyses in the study area showed several statistically significant associations with individual exposure. More specifically, presence of goats within 1000 m was associated with anxiety, allergic rhinitis and number of co-occurring symptoms. In addition, presence of cattle within 500 m was associated with pneumonia; the latter was clearly more prevalent among patients in the study area. Risk of pneumonia was consistently higher in relation to exposure estimates such as presence of goats within 1000 m and distance to the nearest farm, though these results did not reach statistical significance.

Considering the lack of studies on the effects of livestock exposure in this patient group, a direct comparison with prior findings was not feasible. A previous study on patients with COPD (without asthma) living in the same study area showed a comparatively lower prevalence rates of symptoms and infections and no convincing evidence for an association between livestock exposure and health outcomes [30]. Another study [28], showed a higher risk for self-reported respiratory symptoms among individuals with COPD who lived within 500 m from cattle, but that was not statistically significant. In the general population, a higher risk of pneumonia when living in the vicinity of livestock farms is well-documented, but only in relation to goat and poultry [22–25].

A plausible biological mechanism to explain the significant association observed between presence of goats and anxiety is unknown. Nevertheless, people who live in close proximity of goat farms, seem to have a more negative attitude towards farming [39], which might be related to the Q-fever outbreak in the study area several years prior to the study [40]. Concerns regarding the health effects of environmental exposures as well as perceived exposure are well-documented determinants of various clusters of “non-specific” symptoms [41–43], but this is highly unlikely for GP-diagnosed disorders or infections such as pneumonia [39].

For some health outcomes we could verify the risk of living nearby livestock farms. Although it is yet unclear which environmental agents could be responsible for the observed associations, it was recently shown that livestock farms are a source of endotoxins in the areas included in the present study [44]. Moreover, the proxies of livestock exposure that we used were significantly associated with measured endotoxin concentrations [45]. Despite our focus on risk, interestingly enough, a lower prevalence of comorbid conditions was often observed in the control area, and in some cases, in relation to exposure proxies in the study area. Currently the mechanisms of how exposure and other factors interact and develop across time are not well-understood. The analysis described in this article contributes to this lacuna, but further research into these mechanisms is needed to determine which risk mitigation interventions are recommended from a public health perspective.

An important strength of the study was the use of objective outcome data from medical records of general practices, based on a reliable registration system. We also employed two approaches to assess associations; a study vs. control area comparison and use of individual exposure estimates of livestock exposure. In addition, exposure was objectively assessed based on different proxies and information on livestock farm licenses and health outcome data were obtained for the same year.

The cross-sectional design constitutes a study limitation. Second, analyses were not adjusted for covariates such as history of tobacco use and socioeconomic status, since this information was not provided/registered in the EHRs. Nevertheless, previous research has shown that controlling for socioeconomic status or smoking habits did not alter the associations between livestock exposure and health outcomes [28, 46]. Third, considering that this study focused on neighbouring residents and the fact that data on occupational livestock exposure were not available, we excluded patients living within 50 m from a farm. It is therefore possible that not all occupationally exposed people have been excluded, but this probably concerns a small fraction of the sample. A previous investigation in the same study area, using questionnaire data on occupational exposure, showed that only about 2.5% of the residents were living or working on a livestock farm, after exclusion of subjects living within 50 m from a farm [28]. Fourth, only health outcomes were included for which people visit a general practitioner; this could have influenced the prevalence of symptoms, to some extent. Fifth, the lack of a universally accepted case-definition and objective biomarkers of ACOS is an obstacle to the investigation and understanding of its pathophysiology and epidemiology [4, 5, 47]. As a result, estimation of prevalence as well as exacerbation risk is highly depended on the case definitions used in different studies. We therefore employed a rather arbitrary definition that requires further validation. Also considering that this is one of the first studies to explore ACOS comorbidity based on EHR data within the context of environmental health, we cannot ensure comparability with previous research. Finally, a larger sample would increase the study power for the identification of possible exposure-outcome associations.

## Conclusion

In conclusion, this study supports the hypothesis that livestock farm exposures may increase susceptibility to respiratory infections in patients with overlapping diagnoses of asthma and COPD. However, we found no convincing evidence for an association between estimates of livestock density and prevalence of chronic comorbid conditions. Focusing on patient subgroups with different respiratory phenotypes can enhance our knowledge of the risk of environmental exposures in residents with compromised respiratory health.

## Data Availability

In consultation with the Medical Ethical Committee that approved the study protocol, data from the VGO study are not publicly available due to privacy protection of participants. The study’s privacy regulations state that only researchers from NIVEL, IRAS, and RIVM (consortium partners) have access to the study database. Sharing an anonymized and de-identified dataset is not possible as it would still contain Electronic Health Records and the personal data of participants, which could potentially lead to the identification of subjects. Researchers may contact the data access committee through Remco Coppen, PhD, LLM (r.coppen@nivel.nl) or non-personal departmental email (NIVEL: zorgregistraties@nivel.nl) or contact Christos Baliatsas, PhD (c.baliatsas@nivel.nl), Joris Yzermans, PhD (J.Ijzermans@nivel.nl), or Michel Duckers, PhD (m.duckers@nivel.nl).

## Appendix

**Table 6:** Association (OR, 99% CI) ^a^ between fine dust and ammonia emissions from livestock farms and primary outcomes among ACOS patients in the study area

	Modeled emissions from farms			
Comorbidity	Log-weighted fine dust emission		Log-weighted ammonia (NH3) emission	
	Within 500m	Within 1000m	Within 500m	Within 1000m
GERD	1.24 (0.55 – 2.79)	0.96 (0.65 – 1.44)	1.22 (0.52 – 2.88)	1.08 (0.59 – 1.98)
Osteoporosis	0.87 (0.33 – 2.33)	1.1 (0.66 – 1.82)	0.76 (0.26 – 2.2)	1.06 (0.52 – 2.15)
Diabetes mellitus	1.13 (0.62 – 2.06)	0.95 (0.7 – 1.27)	1.11 (0.58 – 2.1)	1.00 (0.65 – 1.55)
Anxiety	0.98 (0.32 – 2.95)	0.75 (0.48 – 1.16)	1.12 (0.36 – 3.46)	0.74 (0.37 – 1.5)
Depression	0.93 (0.44 – 1.96)	0.77 (0.57 – 1.05)	0.84 (0.38 – 1.87)	0.67 (0.42 – 1.08)
Lung cancer	0.37 (0.02 – 5.24)	0.94 (0.35 – 2.5)	0.21 (0.01 – 6.37)	0.83 (0.21 – 3.29)
Hypertension	1.09 (0.67 – 1.77)	1.02 (0.8 – 1.31)	1.01 (0.6 – 1.69)	1.04 (0.73 – 1.48)
Rheumatoid arthritis	0.94 (0.25 – 3.55)	1.06 (0.54 – 2.08)	0.72 (0.16 – 3.18)	0.82 (0.34 – 1.94)
Heart disease (risk) cluster*	0.98 (0.61 – 1.57)	0.95 (0.74 – 1.22)	0.87 (0.52 – 1.45)	0.94 (0.65 – 1.37)
Total prevalence^b^	0.92 (0.56 – 1.52)	0.87 (0.66 – 1.14)	0.79 (0.46 – 1.35)	0.79 (0.52 – 1.18)
Number^c^	0.98 (0.8 – 1.2)	0.96 (0.87 – 1.07)	0.93 (0.75 – 1.15)	0.96 (0.83 – 1.11)
Coexisting symptoms & infections				
Pneumonia	1.5 (0.74 – 3.0)	1.01 (0.71 – 1.46)	1.59 (0.76 – 3.33)	1.04 (0.62 – 1.75)
Sleep problems	0.91 (0.38 – 2.2)	0.9 (0.6 – 1.37)	0.99 (0.39 – 2.5)	0.75 (0.41 – 1.36)
Memory/Concentration problems	i.n.c	0.78 (0.42 – 1.46)	i.n.c	0.53 (0.21 – 1.36)
Upper respiratory tract infections	0.74 (0.42 – 1.32)	0.94 (0.72 – 1.24)	0.75 (0.41 – 1.38)	0.98 (0.65 – 1.48)
Respiratory symptoms	1.04 (0.6 – 1.81)	0.94 (0.72 – 1.22)	0.86 (0.47 – 1.57)	0.87 (0.6 – 1.28)
Dizziness/Vertigo	0.65 (0.18 – 2.37)	1.17 (0.61 – 2.22)	0.62 (0.15 – 2.47)	1.14 (0.48 – 2.69)
Anemia	1.14 (0.5 – 2.6)	0.89 (0.59 – 1.34)	1.12 (0.46 – 2.7)	0.79 (0.43 – 1.46)
Allergic rhinitis/Hay fever	0.84 (0.3 – 2.3)	1.04 (0.65 – 1.68)	0.69 (0.23 – 2.1)	0.93 (0.47 – 1.81)
Total prevalence^b^	0.69 (0.44 – 1.09)	0.9 (0.72 – 1.14)	0.64 (0.4 – 1.04)	0.79 (0.57 – 1.1)
Number^c^	0.92 (0.72 – 1.18)	0.96 (0.85 – 1.07)	0.88 (0.68 – 1.15)	0.91 (0.77 – 1.07)

Abbreviations: *ORs* Odds ratios, *CI* Confidence intervals, *GERD* Gastro-esophageal reflux disease, *i.n.c* Insufficient number of cases

^a^Adjusted for age and gender. OR (99% CI) for an interquartile range (IQR) increase in log-transformed exposure. IQR for ln(fine dust within 500m) = 7.6, IQR for ln(fine dust within 1000m) = 2.4, IQR for ln (ammonia within 500m) = 4.86, IQR for ln (ammonia within 500m) = 2.24

^b^Patients with at least one of the investigated outcomes

^c^Count variables, incidence rate ratios (IRR) are provided

**p*<0.01

## Abbreviations

ACOS: Asthma and COPD overlap syndrome
CI: Confidence intervals
COPD: Chronic obstructive pulmonary disease
EHRs: Electronic health records
GERD: Gastro-oesophageal reflux disease
GPs: General practitioners
IRR: Incidence rate ratios
OR: Odds ratios
PCD: Primary Care Database
